# Offshore wind and hybrids - A counterfactual case study for impacts in the coupled day-ahead market of Europe

**DOI:** 10.1371/journal.pone.0339305

**Published:** 2026-01-16

**Authors:** Felix Jakob Fliegner, Helge Esch, Thure Traber, Marius Schrade

**Affiliations:** 1 Chair of Energy Economics, Faculty of Business Economics, Technische Universität Dresden, Saxony, Germany; 2 50Hertz Transmission GmbH, Berlin, Germany; Technical University of Berlin: Technische Universitat Berlin, GERMANY

## Abstract

Europe’s ambitious offshore wind targets hinge on the development of a single interconnected electricity market, with offshore hybrid interconnectors playing a pivotal role. These interconnectors facilitate both wind energy transmission to shore and cross-border trade, while enhancing market and grid efficiency. This study attempts to quantify these effects through a study of an additional hybrid interconnector in Europe’s present day-ahead electricity market at the example of the Baltic Sea. Using the Euphemia algorithm for single day-ahead market coupling with historical order books from 2023 and 2024, the analysis evaluates power prices, cross-border flows, and economic surplus. It applies a counterfactual “what-if” analysis to real-world market conditions which differs from commonly employed fundamental market modeling. Results reveal that a hybrid interconnector between the Baltic States and Germany would have delivered greater European welfare benefits compared to a radial wind farm connection with an independent parallel interconnector. Notably, price effects and power exchanges extend beyond the hosting countries, underscoring the need for a sea basin-wide planning and cost-sharing approach. Additionally, the distribution of surpluses between offshore producers, transmission system operators and consumers differs between radial and hybrid setups. It highlights the economic complexity and involved risk profiles introduced by offshore (hybrid) assets. This case study confirms theoretical insights from fundamental models with real-life data and identifies key considerations for decision-makers to address distributional challenges and maximize the benefits of offshore hybrid interconnectors in future planning.

## Introduction

The establishment of a single and interconnected electricity market is a central strategy for the European Union (EU) in achieving emissions reduction targets, while maintaining an affordable and reliable energy supply [1]. Electricity generation in offshore wind farms marks an important contribution to this objective [2]. It is motivated by several key factors, including higher capacity factors of offshore wind compared to its onshore counterpart [3], the potential to contribute to a more balanced energy mix [4], and increasing economic viability [5]. In light of these considerations, as well as recent geopolitical developments, the latest commitments from EU member states, Norway, and the United Kingdom have collectively risen to nearly 500 GW of capacity by 2050 [6]. At the same time, the EU highlights the importance of increasing cross-border transmission capacities to facilitate the influx of renewable energy and finds a significant gap between needs and planned investments [7].

In this context, the deployment of hybrid interconnectors, which facilitate both cross-border electricity trade between connected countries and the integration of renewable energy from offshore wind farms, is attracting growing attention in academia [8], industry [9] and among policy makers [10]. The relevance of this topic is emphasized by the fact that much of the offshore infrastructure is yet to be developed, since only 35 GW offshore wind power were commissioned by 2024 [11]. The large-scale development of wind farms and their integration into the interconnected electricity system necessitates a simultaneous uptake of wind farms and interconnections [12]. This allows system planners to trade-off traditional approaches such as radial wind farm connections and independent interconnectors with more integrated solutions such as hybrids and indeed energy islands [13,14].

**Fig 1:**
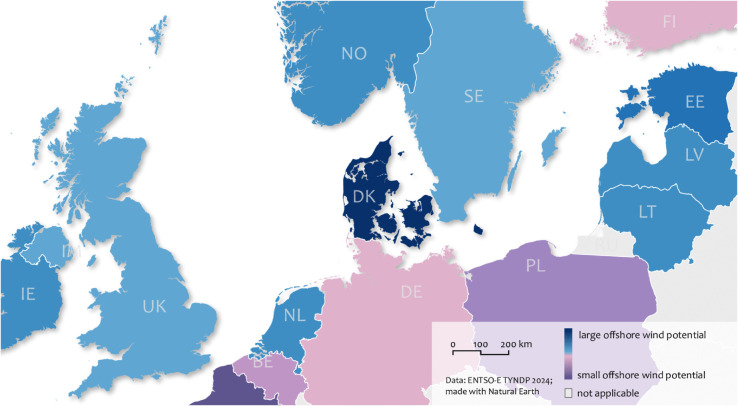
Countries with vast versus limited offshore potentials. Average electric load from TYNDP 2024 [30] (scenario: distributed energy) in relation to the offshore wind power potential according to latest maritime spatial plans and common power densities per country. Base map made with Natural Earth.

In this planning context, hybrid interconnectors have been shown to enhance both market and grid efficiency, while also contributing to reductions in overall system costs and CO_2_ emissions, as demonstrated in several studies [15–18]. Moreover, hybrid interconnectors support the utilization of offshore wind potential that might otherwise remain unexploited. Although Europe possesses sufficient offshore wind resources to meet its development objectives, even under climate change scenarios [19], these resources are distributed unevenly across national jurisdictions [20]. Fig 1 presents a map identifying countries with a structural surplus of offshore wind resources, referred to as “long”. These are defined as countries where the potential power capacity exceeds national demand, even if it was met entirely by offshore wind. Such countries are likely to pursue large-scale deployment only if a simultaneous export pathway to neighboring nations, such as via the power grid, is established. The prospective recipients of this surplus include countries with markedly lower offshore wind potential, designated as “short” on the map. These countries may be compelled to intensively exploit their domestic resources at inefficiently high power densities [21] in order to meet national development targets, a strategy that can intensify wake losses [22,23].

Such inefficiencies can be mitigated, when connecting long countries (i.e. net-exporting) with short countries (i.e. net-importing) [24]. This unlocks additional benefits such as reduced variability of the generation mix and eventually lower residual load due to the decreasing correlation of the wind infeed with larger transmission distances being covered [4,25]. For these reasons, an increasing number of long-distance hybrid projects are currently being investigated in the Baltic Sea [26,27] North Sea [28,29].

### Problem statement and research question

Despite this momentum, the setup of an integrated offshore grid infrastructure involving hybrid interconnectors comes with a set of challenges. A key design question associated to the development of offshore wind farms as part of a hybrid interconnector is their integration into the power market. Historically, offshore wind farms have been regarded as an integral part of an existing bidding zone, commonly denoted as “home market”. An alternative setup involves offshore bidding zones (OBZ), where the wind farm is placed in a separate zone and any connection to adjacent (offshore or onshore) zones is considered as a cross-border interconnection by the market. The latter aims to facilitate the efficient dispatch of the offshore wind farm to respect possible onshore grid constraints for the integration of large volumes of offshore wind generation [31]. The delineation of such bidding zones crucially impacts the economic viability of offshore wind farms being located inside as well as interconnectors being connected to it [32]. Next to the effects within the OBZ, the hybrid interconnector impacts the connecting market areas. Since they are usually located in different countries, the distribution of costs and benefits is a complex undertaking [33,34].

Other challenges include the time interdependency of projects being realized in sequence, incorporating path dependencies for system development [35], impacts on individual project viability [36] and challenges for a forward looking spatial planning for transmission corridors [20]. Not least important, uncertainties remain with respect to technology readiness [37] and the ability of the supply chain to scale up in parallel to the needs [38]. For the sake of brevity they are not further addressed in this work.

Purpose of this paper is to contribute to a better understanding of the key challenges of the introduction of a hybrid interconnector in conjunction with an offshore wind farm being placed in an OBZ. These challenges include the effect on power prices, cross-border power exchanges as well as distributional effects between market areas and market participants (e.g. producers’ and consumers’ surplus). In contrast to the existing literature that looks mainly at long-term future scenarios, this work projects these considerations into the present. The following question is addressed: *What would the power prices and cross-border exchanges have been in 2023 and 2024 if an additional offshore hybrid interconnector had been deployed in the Baltic Sea?*

By answering the research question, three contributions are made:

1. Application of the single day-ahead market coupling analysis to an offshore hybrid interconnector with with historical data as opposed to fundamental modeling.
2. Disentanglement between price and volume effects, alongside the examination of impacts on economic surplus.
3. Focus on the Baltic Sea region, an area less studied than the North Sea.

First, the modeling approach is introduced and followed by a description of the case study of the Baltic Sea region, including the necessary input data and scenario setup. Subsequently, the results are presented and discussed. Finally, the paper concludes with a summary and an outlook on potential directions for future research.

## Materials and methods

For the assessment of the impact of a hybrid interconnector on the electricity market, the European day-ahead market in its current configuration of 2023 and 2024 is studied. According to ENTSO-E 98.6% of electricity consumption is coupled through the Single Day-ahead Coupling (SDAC) in Europe [39]. The following sections briefly introduce the required concepts and the model setup of this study.

### Modeling approach

#### Functioning of the day-ahead electricity market in Europe.

The European day-ahead electricity market operates with zonal pricing, segmenting the internal market into bidding zones. These zones, typically aligned with national borders (except in Denmark, Italy, Norway, Sweden, and the Germany-Luxembourg zone), assume no internal congestion, with constraints occurring only at bidding zone borders [40]. The zonal setup assigns a single market price per bidding zone and market time unit (previously set at 60-minute intervals, with a transition to 15-minute as of June 2025 [41]).

Electricity trade within bidding zones is unrestricted, whereas inter-zone trade is subject to grid capacity limitations. The process of joining different bidding zones to enable transactions that involve selling offers and buying bids from different areas is called market coupling. The capacity limitations to cross-border trades are calculated by transmission system operators (TSOs) for each time-window and capacity calculation region. They apply either the flow-based capacity calculation method, or the coordinated net transfer capacity approach [42]. Some TSOs impose additional allocation constraints beyond bidding-zone borders, such as intertemporal restrictions on high-voltage direct current cables [43]. Grid capacities are determined daily by the TSOs, before nominated electricity market operators (NEMOs) receive these constraints and aggregate market bids until gate closure. NEMOs then consolidate orders into a pan-European shared order book.

The market clearing is achieved through a price coupling algorithm to calculate energy allocation and electricity prices across the participating zones. The algorithm is called Euphemia (Pan-European Hybrid Electricity Market Integration Algorithm), and maximizes the overall economic surplus. At the same time it ensures compliance with grid constraints such that the power flows induced by the executed orders are not resulting in net-positions that would exceed the capacity of all relevant network elements. The algorithm solves one master problem (maximizing economic surplus) and three sub-problems for the price determination and volume. The sub-problems help improve the master problem’s solution by branching and cutting [44].

#### Maximizing economic surplus.

**Fig 2:**
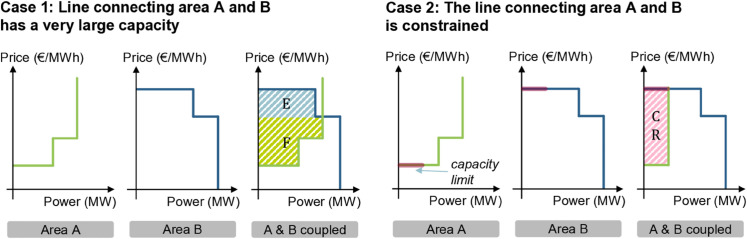
Example of market clearing in two bidding zones. Resembling a hypothetical economic surplus maximization of buying bids (green) and selling bids (blue) in an unconstrained (case 1) and constrained (case 2) market coupling (sketch adapted from [45]).

Fig 2 depicts the maximization of economic surplus and its three components - consumer surplus, producer surplus and congestion rent - in the European day-ahead market. Two bidding zone areas, A and B are assumed, with A comprising two sell bids (green), while area B includes two buy bids (blue). In case 1, sufficient transmission capacity enables full market coupling, leading to a single market with a clearing price equal to the price of the second sell bid. Here, the consumer surplus (area *E*) represents the difference between the maximum willingness to pay and the clearing price, while the producer surplus (area *F*) reflects the difference between the clearing price and the minimum willingness to sell. As there is full price convergence, no congestion rent arises.

In case 2, a transmission constraint limits the trade between area A and B to a maximum power (red line mark), resulting in two different market prices. The price spread represents the marginal value of an additional unit of capacity. The congestion rent for the TSO is thus the area between the green and blue curve denoted as *CR*. Notably, in this case, neither consumer nor producer surplus exists, as market prices align with the respective willingness to buy and sell. For a real aggregated supply and demand curve, refer to the example in Fig 9 of the appendix.

#### Implementation in simulation facility.

This study deploys the Simulation Facility, a tool being maintained by the NEMOs. It allows simulations using historical single-day ahead market coupling data and - as done for this study - user defined inputs [46]. The facility runs the Euphemia algorithm and allows counterfactual analyses through three types of modifications. They include (1) network topology changes, such as interconnector additions or removals, (2) adjustments to network data, i.e. variations to available interconnector capacities, and (3) market data modifications, involving the introduction of new sell or buy bids (resembling generators or loads respectively).

Notice that this approach differs from commonly applied fundamental market or grid models [47] in that this analysis involves the use of actual order books, grid constraints, and market topology from historical production data. It therefore allows the investigation of counterfactual situations as if the market was being operated with an additional offshore hybrid interconnector. This offers a unique opportunity to obtain “real” market prices, and in turn more accurately estimate socio-economic benefit effects disentangled into congestion rents on the transmission asset as well as consumer and producer surplus in the OBZ and the onshore market areas with a minimum set of underlying assumptions. For the workflow of this analysis, it yields the additional benefit that no complex model calibration and validation are required, since it makes use of existing operational data and tools developed and maintained in the European market coupling governance. This makes the approach more lightweight and faster compared to fundamental modeling studies.

While some reviewed work exists on the application of the Simulation Facility in general [48] or the study of OBZ with different models [49–51], this paper is the first one combining both and applying the Euphemia clearing algorithm to a (hybrid) offshore interconnector setup. Only two notable non-scholar exceptions are found in [52], which focused on the technical feasibility to include a wide range of OBZs in the algorithm, and in [53], which conducted a simplified case study for a shorter time period. Another distinction of this work is its temporal perspective, which is routed in a present-day counterfactual analysis as opposed to a long-term future projection towards 2050. The analysis conducted in this study deploys Euphemia as documented in [44] without changes. A mathematical derivation of the involved objective functions and a definition of variables, sets and constraints is found in [54].

#### Limitations of the selected approach.

The selected study setup carries some limits, which require an interpretation of the findings in the appropriate light. For the specific setup of this analysis being a simulation study with a replication of the historical market and grid conditions, only “marginal” changes can be made to the historical data set to yield meaningful results. Hence, only one additional interconnector with a single offshore hub was added, which does not allow for a full quantification of saturation effects that further additional (hybrid) interconnectors or wind farms would impose. This limitation is further discussed in the results section. Moreover, the historical years carry lower levels of renewable energy capacity and interconnectivity compared to a more long-term future scenario of 2030 or 2050. Since the analysis only covers two consecutive years, it is not fully capturing the asset lifetime of around 25 years, which renders the economic viability assessment in this report only an indicative one, as market conditions (such as interconnectivity and renewable energy uptake) will change in the future.

It is further assumed in this study that the entire generated electricity of the offshore wind farm is traded via the day-ahead market. While this is a necessary pre-condition for the applied method to function it is not the only strategy available to offshore wind farms. Common alternative trading regimes are long-term markets (facilitated with contracts for difference) or individual trades over the counter via power purchase agreements. They will be further discussed in the results section of this report. An assessment of different bidding zone delineations or comparison to possible home market setups is also not performed to keep the number of scenarios concise. The authors refer to ref [53] for a discussion on this aspect at a different Baltic Sea example.

Finally, the United Kingdom is currently not part of the coupled power market, meaning it cannot be covered in this exercise. While this might deter single digits in this analysis, it is not likely to change the fundamental findings for the selected model region of the Baltic Sea being presented in this work. It is introduced in the following section.

### The case of the Baltic Sea

The Baltic Sea has historically been, and continues to be, a key region for offshore wind development in Europe [55,56]. By 2050, the three Baltic States—Estonia, Latvia, and Lithuania—collectively target an offshore wind capacity of approximately 14 GW [6], a level of power generation that could surpass the anticipated electrical demand within these countries [30]. In contrast, Poland and Germany, also located within the Baltic Sea region, are expected to face greater electricity demands than can be met by their domestic (offshore) renewable energy resources by mid-century [57,58]. This discrepancy (which is also being illustrated in the map of Fig 1) underscores the importance of examining the region as a case study for long-distance cross-border collaboration in offshore wind energy development.

#### Scenarios and input data.

Inspired by recent developments in the region to further interconnect Germany and the Baltic States [59], three counterfactuals are simulated with a 2 GW offshore wind farm located in the eastern Baltic Sea. It is integrated through a (new) OBZ that is connected via three different topologies:

1. **radial:** A 2 GW radial connection to Germany
2. **interconnector:** A 2 GW radial connection to Germany plus a parallel 0.5 GW interconnector from Germany to the Baltic States.
3. **hybrid:** A hybrid connection with a 2 GW leg to Germany and 0.5 GW leg to the Baltic States.

The assumed technical setup is in agreement with the European trend towards a more standardized design of offshore wind farms, involving wind farm sizes of 2 GW each [6,60]. The onshore grid connection is assumed to be realized with high voltage direct current (HVDC) assets given the long distance that needs to be covered and the large power capacity involved [61]. The asymmetrical transmission capacity of the hybrid interconnector with a smaller leg towards the east is motivated by the considerably smaller size of the power market of the Baltic States as well as grid limitations with respect to the connection of large generation capacities (e.g. limitations imposed by the Latvian TSO AST outlined in ref [62]).

**Fig 3:**
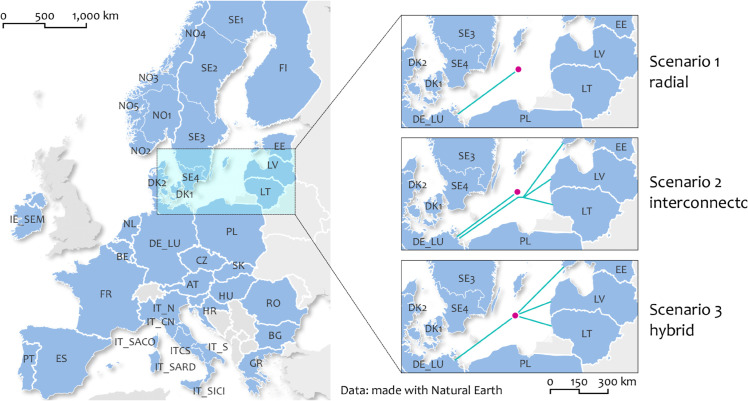
Perimeter of the study. Map showing the coverage of simulated market areas along with the approximate location of the modeled offshore hub in the Baltic Sea. Own depiction with data from [63], base map made with Natural Earth.

The simulations are carried out for the entire European single day-ahead market at a zonal resolution (i.e. market areas) as illustrated in Fig 3 (refer to Fig 11 in the appendix for a more detailed topology representation of the interconnected bidding zones). The study setup gives preference to an agnostic topology design with respect to the exact landing area of the hybrid interconnector in the Baltic State region. Since the generation capacity of the new offshore hub exceeds average (and peak) demand of either of the three Baltic States, the integration of the additional offshore hub must be distributed across these markets to prevent an immediate saturation of the market zones that would skew all other findings (c.f. Table 4 in the appendix for illustration). For this reason the northern part of the interconnector is split into three equally sized legs of 167 MW each, to link all Baltic States equally (c.f. green lines in Fig 3). This should not be interpreted as a multi-terminal grid setup but serves merely as a calculation help for the market coupling. While this establishes additional trade opportunities within the Baltic countries in theory, the simulation results show that this internal trade via these three additional legs averaged at 2 MW, which is considered negligible for the purpose of this analysis.

The offshore wind farm is represented as a single generator located within a newly established OBZ. This modeling approach aligns with recent developments in the Baltic Sea, such as the creation of a new OBZ for a prospective offshore hub on the island of Bornholm [64]. In addition, it can be demonstrated with the simulation results in the following section, that a home market integration of the additional offshore hub would yield the same results as computed for scenario 1 already. This equivalence arises from the fact that the transmission capacity available on the relevant line is equal to or greater than the maximum wind generation capacity. Consequently, prices within the OBZ consistently converge with those in the onshore bidding zone of Germany.

This case study covers the period from 2023 to 2024, with results then being shown as normalized values for a single year to smoothen out potential non-typical conditions. The required weather data for the additional offshore wind farm is based on measurements for the same period in the Kriegers Flak offshore wind farm (located in the Baltic Proper between Denmark, Sweden and Germany) and scaled linearly to the new capacity being used in this study. While this neglects possible de-correlation effects between southern and eastern Baltic Sea wind farm locations, it preserves the hourly fit of actual measured wind profiles to the historical order books. The resulting wind profile shows typical seasonality, with higher capacity factors in winter and lower in summer, averaging at 45% (c.f. Fig 10 of the appendix).

The wind farm generator is added to the order books with a fixed bid of 5 €/MWh for each hour of the year where the wind availability is sufficient for power production. The lifetime is assumed for 25 years, which scales the discounting of investment and operational costs as shown in Table 1 accordingly. The parameters are aligned with and taken from the Ten-Year Network Development (TYNDP) version 2024 [30].

**Table 1:** Parameters and input data for the case study.

Financial Parameters		Distances		
Offshore wind farm	2,060,000 €/MW	Scenario 1	600 km	at 2,000 MW
	65,000 €/MW/a	Scenario 2	600 km	at 2,000 MW
Transmission system	1,617 €/MW		650 km	at 500 MW
	40 €/MW/a	Scenario 3	600 km	at 2,000 MW
WAAC real	5%		100 km	at 500 MW
Lifetime	25 years			

## Results

Offshore wind generation impacts the coupled energy system beyond the countries which are directly connected. Purpose of the following section is to disentangle these distributional effects regarding prices, power exchanges and socio-economic benefit. The section then proceeds with a discussion on the related challenges and (economic) risks in context of the studied setup.

### The builder does not take it all

#### Price effects.

**Fig 4:**
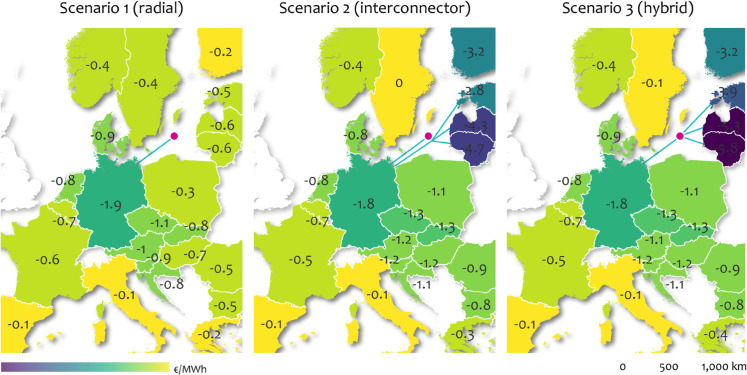
Volume weighted price effects associated with an additional offshore hub in the Baltic Sea in three scenarios. Left: Offshore hub with a 2 GW radial connection to Germany. Middle: Offshore hub with a 2 GW radial connection to Germany plus a parallel 0.5 GW interconnector from Germany to the Baltic States. Right: Offshore hub as part of a hybrid interconnector with a 2 GW leg to Germany and a 0.5 GW leg to the Baltic States. Own analysis, base map made with Natural Earth.

The simulation results presented in Fig 4 depict the reduction in the weighted average of day-ahead power prices over the studied period. The color gradient illustrates the price impact of integrating an additional offshore wind farm into the market, highlighting a significant decrease in prices that extends well beyond the directly connected (i.e. hosting) market areas.

In the first scenario, where the 2 GW offshore hub is connected radially to Germany, price reductions of up to 1.9 €/MW are observed within Germany, with smaller yet discernible effects in neighboring regions. A similar pattern is evident in the second and third scenario, where the Baltic States are linked via a conventional interconnector (scenario two) or integrated into a hybrid interconnector (scenario three). Although the price reductions in these latter scenarios are slightly lower (up to 1.8 €/MW in Germany), the effects are distributed more extensively across the continent. The Baltic States experience the largest price deltas in this simulation, for a significant displacement of high-cost thermal generation in the merit order by the integration of offshore wind via the conventional interconnector (scenario two) or hybrid interconnector (scenario three). The observed movements are in agreement with a recent analysis conducted by [65] that finds weighted average price effects between −1..−4 €/MWh for a difference in developing offshore wind involving hybrid interconnectors versus the lack of it.

**Fig 5:**
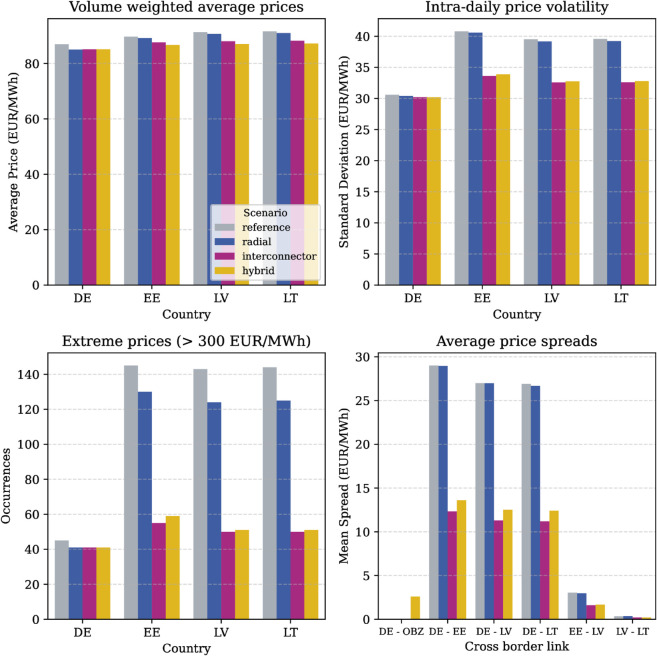
Price effects overview for selected countries. Summary of selected price indicators for the market regions of Germany, Estonia, Latvia and Lithuania across all simulated scenarios and in relation to historical values. Intradaily standard deviation is the mean of all 24-hourly standard deviations (i.e. one value per simulated day).

Looking beyond average prices, this simulation confirms the expectation that additional interconnection and wind generation capacity in the system reduces price peaks, standard deviation as well as spreads between market areas. This is especially true for the directly connected market areas of Germany and the Baltic States. These effects are summarized in Fig 5.

Maximum observed prices drop by up to 55% in the third (hybrid) scenario in Latvia and Lithuania respectively (with 41% in Estonia). This can also be measured in an up to 65% decline of hours with extreme prices (here, defined as prices above 300 €). In conjunction with lower average prices, this signals a structural change in market conditions (e.g. through additional renewable infeed at low marginal costs and displacement of expensive peak generators in the merit order).

A closer look into the daily price volatility expands this notion for the shorter time horizon. In this analysis daily price volatility is defined as the average of all individual 24-hourly standard deviations for each simulated day. In the interconnector and hybrid scenario, the daily standard deviation is reduced by 17% a movement that can also be found in a comparable day-ahead market simulation for a hypothetical setup of different offshore hybrid interconnectors [66]. This reduction shows how the market clearing algorithm uses the additional flexibility of the interconnector every day, which lifts the operational constraints the system is operating in. As a result of this active usage of this flexibility, the mutual price spreads among the directly connected market areas of Germany, Estonia, Latvia and Lithuania are approximately halved compared to historical levels.

The (hybrid) connection of offshore wind to shore can, therefore, contribute to a more harmonized, single European electricity market, while improving price stability as envisaged in [1]. This is not only valuable for average periods as studied above. Instead it is heightened importance during periods of crisis, or at other times when prices are high. The analysis results reinforce the sentiment of previous studies that (hybrid) interconnectors and the integration of offshore wind contribute to security of supply in a positive way by strengthening Europe’s internal energy market and lowering price variability [67,68]. The marginal differences in price effects between scenario two and three (denoted by very similar yellow and red bars in the bar plots) reveal, that - from a market price perspective - it is not of paramount importance which topology is chosen, as long as the effective cross-border trade capacity is available to the market.

#### Cross border trades.

**Fig 6:**
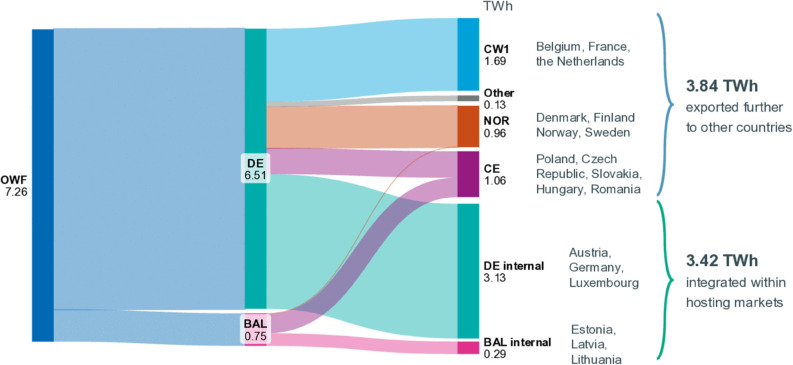
Propagation of electricity injection from the offshore hub into the hosting markets and beyond. Average annual values for the simulated two-year period for scenario three grouped into macro-regions as per TYNDP [69].

The price effects induced by the additional offshore generation and connection in this simulation are due to additional trade volumes and an already well-coupled continental electricity market. Fig 6 illustrates for the hybrid scenario (number three) that the directly connected market areas of Germany and the Baltic States increase their net exports by a total of 3.84 TWh compared with the actual trades carried out in 2023 and 2024 (reference case). This represents 53% of the total electricity supply of the additional offshore hub in the Baltic Sea (7.26 TWh) and illustrates, how markets that are not directly linked to hybrid interconnectors benefit from their additional offshore injections.

Given the size of the power market in the three Baltic States, with an average annual net power consumption of approximately 27 TWh in 2023 and 2024 [70,71], the introduction of an additional 2 GW offshore wind farm (generating 7 TWh annually) represents a significant change to the region’s energy mix. Despite all three countries maintaining a renewable energy quota exceeding 50%, the scenarios analysed for the years 2023 and 2024 still incorporate a substantial proportion of fossil fuels within the energy mix, amounting to 4.6 TWh in 2024. The newly introduced offshore generator will partially displace this fossil fuel consumption. This Merit-Order effect is a key contributor to the observed reductions in electricity prices. If the analysis were extended beyond 2030, price effects would be diminished due to the anticipated increase in onshore renewable energy sources and the ongoing expansion of cross-border capacity planned for the region [30].

Consequently, the observed price effects and cross-border trades in this study are likely to reach a saturation point with any further additions of offshore generation and interconnector capacity, and such trends should not be extrapolated for future renewable generation capacities in the region. The analysis conducted in ref [72] on the sequential installation of two interconnectors between the United Kingdom and France provides evidence of this movement. Given the specifics of the selected modeling framework, this article refrains from simulating diminishing returns for additional interconnector or generation capacity. For a more comprehensive discussion on this matter, the authors direct readers to existing scholarly work addressing price cannibalization effects [73] and the welfare interdependence of alternative interconnectors within a sea basin [74].

#### Utilization rates.

**Fig 7:**
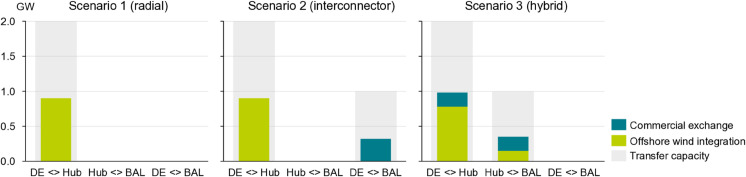
Average utilization of transmission assets. Average commercial exchange between BAL (Estonia, Latvia and Lithuania combined) and DE (Germany) and offshore wind integration from the offshore hub in relation to the rated transfer capacity.

In addition to facilitating increased cross-border trade, the implementation of the hybrid interconnector is shown to enhance the utilization rate of offshore transmission assets compared to the radial scenario. When the offshore wind farm is connected via a radial link, the asset utilization rate aligns with the capacity factor of the wind farm, which is approximately 0.9 GW, or 45% of the installed capacity (c.f. left plot in Fig 7). This utilization rate cannot be further increased, as the cable is solely dedicated to linking the wind farm to shore. A similar observation is found for scenario two, where the wind farm is still connected radially to shore. The parallel interconnector to the Baltic States shows a utilization rate of 30%.

The hybrid scenario combines both the commercial exchange and offshore wind integration in both connected market areas. As illustrated in the right plot of Fig 7, this dual functionality results in an increase in cable utilization to 49% along the southern leg to Germany. The northern leg, which links to the Baltic States, operates at a smaller absolute transmission level due to the reduced capacity of the link (0.5 GW), but its utilization rate is comparable to that of the interconnector in scenario two. Furthermore, a transit flow is visible, which represents the commercial exchange between Germany and the Baltic States that is realized via the offshore hub. It describes a subtle seasonality, with the majority of commercial exchanges towards the Baltic States occurring during summer months (during peak solar PV injections in Germany) and stronger exchanges towards Germany in winter, when wind availability is higher (c.f. Fig 10).

The proportion of transmission capacity used for cross-border flows is significantly greater for the Baltic States link than for the German one. This observation underscores the point that hybrid interconnectors may not always be used symmetrically, adding complexity to the evaluation of their benefits and the equitable distribution of costs associated with such assets.

It should be noted that not all similar (hybrid) offshore connections would lead to the same price and cross-border trade effects. However, the analysis clearly demonstrates that such projects almost certainly affect non-hosting countries, i.e. neighboring market areas around the same sea basin and beyond. This underscores the complexity involved in realizing such transmission systems and sharing out their costs, benefits and risks. The next section deepens this observation by assessing the hypothetical economic case of producers and consumers as well as the operator of the transmission asset.

### Economic viability

The price effects (c.f. Fig 4) and commercial power exchanges (c.f. Fig 6), both reaching beyond the hosting countries, provide a first indication of possible benefits of this additional offshore hub. With Table 2 it is confirmed that the overall effect on the socio-economic welfare (SEW) of all three assessed topologies is indeed positive. The largest driver behind this is the integration of additional 2 GW offshore wind generation, since the wind farm addition, even when integrated radially without additional interconnection (scenario one), already yields a total SEW increase of 17,000 M€ annually (in comparison to the reference case with unchanged historical order books). Additional interconnection increases this welfare effect only slightly with the delta being largest for scenario two, involving the largest degree of freedom for the optimizer to dispatch commercial exchanges between Germany and the Baltic States compared to the hybrid setup (scenario three). The negative producer surplus across all scenarios can be explained through a displacement of thermal generators by the additional offshore wind generation at lower marginal costs (i.e. lower bids in the order books).

**Table 2:** Total socio-economic benefit change in Europe and project costs.

M€ p.a.	Scenario 1 radial	Scenario 2 interconnector	Scenario 3 hybrid
producer surplus	−3600	−4700	−5000
consumer surplus	9200	11,700	11,900
congestion income	11,400	12,500	11,500
total	17,000	19,500	18,400
annualized project costs	6000	6500	6200

Delta of producer and consumer surplus and congestion income summed across all simulated market areas for the three scenarios in comparison to the reference case as well as annualized project costs (CAPEX and OPEX).

**Fig 8:**
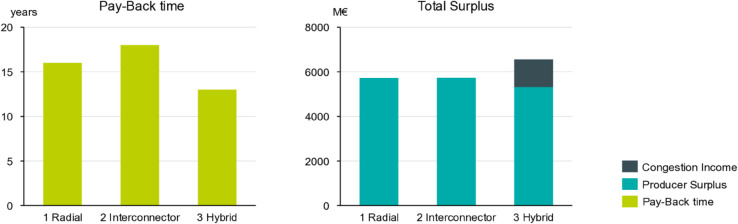
Pay-back time and total surplus in the offshore bidding zone. Approximated time for the investment costs of the offshore transmission assets and the offshore wind farm to break-even with the extrapolated surplus within the OBZ.

When comparing the welfare increase to the annualized investment and operational costs for the three scenario variants (last row of Table 2), it is found that this project would have been economically viable for the European market as a whole under these historical conditions. Even when focusing on the offshore bidding zone, i.e. only accounting for the welfare gains of this particular market area and not the entire union, this finding holds true as indicated by the bar plots in Fig 8. With a pay-back time of 8 to 18 years across all three scenarios, the project would have recovered its costs well within the assumed project lifetime of 25 years only with the producer surplus and congestion income of this offshore bidding zone alone.

Notice that under this metric, the second scenario is no longer the best performing one (in contrast to the total European SEW perspective taken in Table 2), but instead the hybrid one. This is due to the much higher investment costs for scenario two that would require to build parallel power lines for several hundred kilometers to obtain the topology as sketched in Fig 3. In other words, the extra gain in welfare is offset by much higher costs. Although the hybrid scenario is best performing from a portfolio point of view, the producer surplus of the offshore wind farm is lowest in this case (c.f. bar plot on the right in Fig 8); a finding that is in agreement to a similar analysis of a hypothetical hybrid interconnector between Finland and Sweden in ref [53]. This leads to less favorable financing conditions for the wind farm investor. As will be discussed in the following section on price risks, this is a key challenge for such integrated grid and wind farm projects, that different actors such as TSOs and wind farm developers benefit very differently from hybrid offshore topologies and OBZ market setups.

While the price effects observed in this analysis suggest that the wind farm and transmission infrastructure could recover their costs within the expected lifetime of the assets, it is evident that actual investment decisions cannot be based on a single case study. Clearly, this exercise is hypothetical, and interconnectors commissioned in the coming years will encounter different market conditions and boundary constraints. Moreover, the studied interconnector setup will not face stable market and grid conditions throughout its existence as further interconnectors and renewable generators will enter the market and change the boundary conditions. Nevertheless, the simplified analysis serves as a useful illustration of key risks and challenges associated with the development of offshore wind through hybrid interconnectors. They concern price risks (i.e. the uncertainty of fluctuations in electricity prices that can affect the revenue and profitability of offshore hybrid projects [75]), volume risks (i.e. the uncertainty in the quantity of electricity generated or traded compared to expected levels, regardless of price changes [75]) and market setup risks including changes in grid topologies, and the delineation of market areas [76]. They are discussed further in the next sections along with a brief overview of possible mitigation measures.

#### Price risk.

One form of price risk arises from the structurally lower price level in an OBZ compared to the home market [77]. In most cases, the connection to the higher-priced market area is the first to experience congestion. As a result, the electricity price in the OBZ tends to align with the market area where the hybrid interconnector remains uncongested. This convergence leads to a downward pressure on prices within the OBZ, thereby negatively affecting the producer surplus of offshore wind farm developers as demonstrated in the simulation (c.f. Table 2). During periods when export capacity to adjacent market zones is constrained, prices in the OBZ may even fall to zero, as the offshore generator becomes the sole bidder. Evidence of such occurrences is reflected in the price spreads observed in the hybrid scenario analyzed in this study (see Fig 4). Additionally, under flow-based market coupling, price formation within the OBZ can yield non-intuitive outcomes, including prices that fall outside the range of adjacent bidding zones. These anomalies arise when trades involving the OBZ influence critical network elements elsewhere in the system, and the marginal value of adjusting offshore wind generation diverges from the economically optimal solution [78].

Next to the average level, price spreads across distant locations can lead to challenges in direct marketing of the generated electricity. Consumers being located onshore (e.g. in Germany or the Baltic States) will often be exposed to a structurally higher price compared to the OBZ which makes the establishment of long-term contracts such as power purchase agreements or contracts for difference unattractive as high risk premia would be involved [79].

Other more general price risks involve the uncertainty of future commodity price levels (which directly impact the bids of thermal generators in the merit order) or capture prices related to weather conditions, commodity price volatility and unit availability [80]. As they are not particularly heightened in the context of this case study they are not further discussed in this paper.

#### Volume risk.

Within the flow-based capacity allocation procedure, the OBZ competes with all other bidding zones, both onshore and offshore, for limited transmission capacity. Allocation decisions are determined by the Euphemia optimization algorithm, which is designed to maximize the economic surplus across all participating markets. If transactions in other zones, whether onshore or offshore, or cross-border exchanges including transits through the OBZ (as illustrated in Fig 7 for scenario three), generate greater economic benefit, the OBZ may receive a smaller share of the available transmission capacity. This can result in unfulfilled sell orders, given the relatively small size of the OBZ and the absence of local demand compared to onshore market areas [81].

It may also occur that the total generation capacity of the offshore wind farm cannot be fully absorbed by the onshore grid, which is subject to constraints across the entire network. In such cases, the maximum net position of the OBZ is limited by the available cross-zonal capacity, potentially leading to curtailed generation volumes. Since both cross-zonal transmission lines and critical onshore transmission lines define the boundaries of the flow-based domain, constraints in the onshore grid can also restrict export capacity to levels below the total connection capacity of the offshore wind farm [76]. This effect is particularly pronounced in situations where the offshore wind farm is “overplanted”, meaning its installed generation capacity structurally exceeds the transmission capacity of the links to adjacent bidding zones. However, this does not necessarily increase the risk exposure, as such a structural transmission cap is easier to anticipate.

General unavailability of the transmission asset, for example due to unplanned outages or delays in commissioning, can also impose a volume risk for the offshore generator. In the case of a single radial connection, this risk may result in a complete loss of export capability. A hybrid configuration introduces a degree of redundancy, as a second export route exists to enable partial export of the available power. Although this risk is present in both home market and OBZ settings, the implications for compensation and hedging strategies differ.

#### Market setup risk.

The sequence of scenarios in the case study in this paper is an example for an additional risk which an already existing wind farm can be exposed to. It involves changes in market regimes and grid topologies during the lifetime of the generation and transmission asset which can deter the economic case of the initial investment. As transmission capacities are expanded (e.g. via the installation of another parallel interconnector in scenario two or an additional link to an existing offshore hub in scenario three), power prices in OBZs are susceptible to changes. Such a risk is also present when an existing wind farm’s connection type is altered from a (home market) radial to a (offshore bidding zone) hybrid interconnector setup [82].

Notice that the derived risks are not exclusively known for the context of (hybrid) offshore wind connectors, since any large energy infrastructure investment with high upfront capital expenditures is exposed to them [83]. They are, however, elevated in an offshore context as investment decisions must be taken amidst changing regulatory conditions, which exacerbate project risks and ultimately financing costs. Examples for such changes are a possible introduction of future OBZ that do not exist in today’s market regime, or future revisions of their delineation. Regulatory countermeasures could entail formal assurances by authorities or governments to refrain from implementing retroactive changes that may compromise investor confidence [84].

#### Home market versus offshore bidding zone.

Market regime changes are particularly significant for offshore wind farms connected either radially (scenario one) or hybrid (scenario three), as both face the aforementioned risks. In the radial case, integration into the home market can mitigate these risks by enabling direct trading with local demand, akin to a diverse mix of onshore orders, and resolving congestion issues post-market closure without loss to the generator (e.g. through redispatch or counter trade).

Conversely, generators in an OBZ are more vulnerable to these inherent market risks, which encompass not only uncertainties regarding price spreads and volumes but also the practical (i.e. contractual) management of these differences. Consequently, system planners and policymakers must balance the societal benefits of offshore (hybrid) interconnections against the commercial risks and complexities involved in their implementation. The following section outlines several relevant considerations.

### Discussion of risk mitigation measures

Mitigation measures against the identified price and volume risks involve power purchase agreements (PPA), contracts for difference (CfD), long-term transmission rights (LTTR), transmission access guarantees (TAG) as well as technological measures. Their capability in mitigating the aforementioned risks is summarized in Table 3 and elaborated in the upcoming sections.

**Table 3:** Economic risks and impact of different mitigation options for the realization of offshore hybrid interconnectors.

Risks Measures		PPA	CfD	LTTR	TAG	offshore load	grid buildout
Price	Structurally low	↓	↓	↓	⇌	↓	⇌
	Non-intuitive	↓	↓	↓	⇌	↓	⇌
	Locational spread	⇌	↓	↓	⇌	↓	⇌
Volume	Unavailability	⇌	⇌	⇌	⇌	↓	↓
	Cap. calculation	↘	↓	⇌	↓	↓	↓
	Cap. allocation	↘	↓	⇌	↘	↓	↓
Market setup	OBZ delineation	⇌	↓	⇌	↓	↓	⇌

Simplified illustration of mitigation measures with no definitive (⇌), somewhat reducing (↘) and mostly reducing (↓) effect on the risk categories. CfD assumes a two-sided capability based CfD setup. LTTR assumes a financial transmission right (as opposed to physical). Based on and expanded from: [66].

#### Power purchase agreements.

PPAs are a key financial hedging tool in power markets, binding consumers to purchase the output from energy producers. They enable market participants to manage risks bilaterally, thereby avoiding legislative interventions. These long-term contracts can be classified into physical PPAs, which involve direct delivery, and financial or virtual PPAs, which rely on differential payments based on market reference prices [85].

In the context of offshore wind farms within OBZs, PPAs can be tailored to mitigate specific risks, such as structurally lower or even collapsing prices. In doing so, they improve project bankability and serve as a commercial instrument for reducing risk exposure in merchant markets [86]. However, challenges arise from the price spread risk (as found in the hybrid scenario, c.f. Fig 5), which complicates agreements between offshore generators and onshore demand agents. In addition, the limited practical experience with hybrid projects may result in excessive risk allocation to offshore wind farms [82].

#### Long-term transmission rights.

LTTRs can be either physical or financial. Financial LTTRs (also abbreviated as FTRs) are most commonly used in Europe and serve to mitigate locational price spread risks in electricity markets by allowing participants to secure the price difference between two neighboring bidding zones, including but not limited to OBZ [87]. In doing so, they address a key limitation of the aforementioned PPAs. When market prices differ from the contracted price, financial LTTR holders are compensated based on the contracted volume multiplied by the price difference. This mechanism can redirect congestion income from TSOs, who typically auction LTTRs, to offshore generators, thereby transferring the economic surplus observed in the OBZ back to producers [51].

However, LTTRs do not mitigate volume risks, as compensation is only provided for electricity that is actually sold. In addition, the FTR mechanism must be carefully designed to ensure that it adequately addresses operational deratings and price collapse risks associated with hybrid assets in the OBZ [87]. A broader limitation of FTRs and forward markets lies in their restricted time horizon. Most trading in EU forward markets currently takes place only a few years ahead [88], which is insufficient for covering investments such as hybrid interconnectors or wind farms that require visibility over several decades. The issuance of physical LTTRs could serve as a countermeasure to improve the security of long-term trades and enhance liquidity in this market segment.

#### Contracts for difference.

In electricity markets, CfDs typically involve long-term contracts between electricity generators and a central counterparty (e.g. the government), providing stable revenue streams for generators. When market reference prices fall below the agreed strike price, the generator receives compensation for the difference, while reimbursement is made to the regulatory body when market prices exceed the strike price [89]. Various CfD designs exist, with the two-sided capability-based CfD being most relevant in the offshore context [86,90]. This design guarantees a fixed price based on the potential rather than the actual electricity injection, thereby covering both price and volume risks by ensuring payments are made based on potential production capacity.

Although this approach offers predictable revenue streams and reduces financing costs, thereby encouraging investment in hybrid offshore wind farms, it also presents potential drawbacks. Widespread adoption of CfDs may affect the functioning of other markets and instruments used for hedging, such as PPAs and LTTRs [91]. There is also a risk that both the strike price and the long-term reference price may be set inefficiently, which could lead to market distortions. These include windfall profits if strike prices are set too high, or reduced renewable investment if reference prices are set too low, ultimately resulting in a decline in net socio-economic welfare [92].

#### Transmission access guarantees.

The primary objective of TAG is to mitigate volume risks by ensuring that the total export capacity of the OBZ matches the available offshore generation capacity [87]. In situations where this cannot be guaranteed, compensation is provided to the generator. This is calculated based on the price spread between the reference bidding zone and the OBZ, multiplied by the total available offshore generation. The compensation is funded through congestion rent and is paid by the TSO responsible for the limiting network elements. The intention is to incentivize TSOs to restrict capacity only when the overall system benefits outweigh the opportunity costs incurred by offshore generators.

Although TAG can contribute to revenue stability for offshore generators, there is uncertainty regarding whether the congestion income raised for compensation is sufficient and reliable, given its inherent variability and the potentially conflicting priority objectives for its allocation as defined by regulators [93]. It is also subject to debate whether TAG distorts competition, as developers located in OBZs under TAG may be unfairly shielded from competitive market forces and, in effect, receive preferential treatment compared to competing cross-border flows [94].

#### Other measures.

More generally, technological changes in the offshore grid can facilitate the aforementioned measures. The first one involves the connection of additional demand to offshore generation hubs (e.g. through offshore electrolysis [14], electrification of oil and gas platforms [95], CO_2_ sequestration facilities, sub sea data centers [96], storage [97] or power supply for aqua cultures [98]). It can make OBZs less sensitive to flows on adjacent borders and would create more possibilities to utilize power during low or zero price periods and mitigate price and volume risks in OBZs effectively [99]. In the Baltic and North Sea region such measures are likely to be implemented in the future for the widespread existence of legacy infrastructure of the oil and gas sector and future visions of offshore hydrogen infrastructure [100,101].

A strategical and planning mitigation measure denotes an increased interconnection of several OBZs among each other and to several onshore bidding zones. This improves the redundancy of infrastructure availability and trading alternatives. Such an increased interconnection of offshore hubs and bidding zones (potentially involving several countries) does, however, increase the complexity with respect to financing, funding and the allocation of benefits across the involved markets [33,34]. Ref [102] argues that joint sea basin planning with a bundled assessment of projects will be required to overcome this barrier. Eventually this would require a more holistic and bottom-up planning approach compared to the more national and case-by-case procedure of today. Furthermore, it has been suggested in the public debate [84,103] that, once project portfolios are identified which involve the coordinated development of interconnectors and wind farms, project specific ownership structures may be established. They are sometimes referred to as “special purpose vehicle” and would facilitate financing, installation and operation, while also aiming to balance the risk exposure among the participating entities and to ensure a fair allocation of costs and benefits. A detailed assessment of such frameworks is recognized as a relevant subject for future research.

## Conclusion and outlook

This paper presents an analysis of the European coupled day-ahead market, assessing the impact of an additional offshore wind power hub in the Baltic Sea. A counterfactual analysis of historical order books is applied using the Simulation Facility tool, offering a unique opportunity to calculate actual market prices and evaluate changes in commercial exchanges and overall economic surplus under three distinct scenarios. While the case is studied for the Baltic Sea region, similar movements may be observed in other sea basins with comparable boundary conditions, such as the North Sea.

The findings highlight how even modest changes to network topology and market data can have widespread effects, and reveal the dual nature of benefits and drawbacks associated with such projects. All three scenarios are found to be beneficial from a European societal perspective (i.e. contributing to lower electricity prices, reduced spreads and volatility as well as enhanced system efficiency), yet distributional challenges emerge both geographically across country borders and within the market among different participants.

The first challenge concerns the potential asymmetry in cost-benefit allocation. As benefits extend beyond the hosting countries and the immediate sea basin, there is a clear need to consider mechanisms for redistributing value from beneficiary markets to hosting regions that bear the costs. Addressing this imbalance is crucial to ensuring the economic feasibility of hybrid offshore wind projects. Future research should explore policy frameworks that mitigate these disparities at the sea basin level. In addition, policy makers need to support the establishment of equitable investment conditions, thereby enhancing the long-term viability of such projects within an evolving energy landscape.

The second challenge relates to distributional effects along the energy value chain (i.e. among generators, transmission and consumers). While the hybrid scenario exhibits superior overall performance compared to the radial configuration, it further suppresses producer surplus, both onshore through the displacement of thermal generation bids, and offshore through structurally lower prices within the OBZ. This intensifies a range of price and volume risks and suggests a potential conflict of interest in system development. Any measure that seeks to address these risks will need to balance societal benefits of offshore wind deployment with the economic viability for individual developers. Further research could provide deeper insights into these trade-offs by examining the impact of alternative market zone delineations and market designs on the resulting optimal build-out of offshore infrastructure.

Offshore wind plays a significant role in the coupled electricity market of Europe, and this study demonstrates that its influence is not limited to long-term future scenarios but is also evident within the present-day electricity market. Despite the high upfront costs and the complexities involved in sharing them and aligning the benefits accordingly, the timely deployment of such projects will be essential for Europe to advance towards its offshore wind capacity targets and broader climate objectives.

## Appendix

### Brief mathematical representation of the optimization problem being implemented in Euphemia simulation facility

The market clearing mechanism of the single Day-Ahead spot market for electricity in Europe is optimized under the objective to maximize the economic surplus under a set of constraints such as supply and demand balance, network transmission constraints, producers’ capacity constraints and ramping constraints. The complete list of constraints and a distinction of various order types is found in the official documentation in [44]. The following mathematical representation of this optimization problem highlights the most important part of it to keep the description concise and brief.

With Euphemia, the market outcome is obtained by...

1. … solving a primal problem which maximizes economic surplus across all bidding zones, subject to network constraints. The variables are the accepted orders (expressed in quantities and quantity-prices for bids and offers respectively) and cross border flows. Constraints apply for the precedence of orders as well as network load limitations.
2. … solving the dual problem which optimizes market clearing prices and congestion rents being constrained on the price differences.

The primal problem reads (adapted from [54]):

$$\max_{x}\;\sum_{z\in 𝒵}\left(\sum_{b\in {ℬ}_{z}}Q_{b}^{z}P_{b}^{z}x_{b}^{z}-\sum_{o\in {𝒪}_{z}}Q_{o}^{z}P_{o}^{z}x_{o}^{z}\right)$$ (1)

$$\text{s.t.}\;\sum_{z}\left(\sum_{b\in {ℬ}_{z}}Q_{b}^{z}x_{b}^{z}-\sum_{o\in {𝒪}_{z}}Q_{o}^{z}x_{o}^{z}\right)=0$$ (2)

$$M_{p}Ex\leq b_{p}$$ (3)

0≤x≤1 (4)

Here, 𝒵 is the set of bidding zones with ${ℬ}_{z}$ and ${𝒪}_{z}$ being sets of bids and offers per zone respectively. The vector *x^z^* contains all variables describing what fractions of quantity-price pairs ($Q_{o}^{z}$, $P_{o}^{z}$, $Q_{b}^{z}$, $P_{b}^{z}$) are accepted in a given time step. The power balance per zone is enforced via eq. (2) to ensure that demand equals generation. Network constraints are enforced via eq. (3) to keep the power exchange not larger than the remaining available margin (RAM) for each critical line (*b*_*p*_), where *Ex* is the vector of all net-zonal generations and *M*_*p*_ a vector of line-zonal power transmission distribution factors (PTDFs) which quantify the effect of a one-unit change in zonal power on relevant transmission lines (critical network elements).

Once the primal problem is solved, the dual problem can also be solved to obtain marginal transmission costs and eventually zonal prices (c.f. ref [54] for theorems and proofs required for this).

**Table 4:** Average and peak demand of the Baltic States in the study period.

MW	Estonia	Latvia	Lithuania
average	830	730	1150
peak	1500	1150	2000

Excerpt of the single day-ahead market coupling matched demands from the simulations for both studied years to illustrate the market size and put the generation capacity of the offshore wind power hub into perspective of each of the connected Baltic markets.

### Additional figures

**Fig 9:**
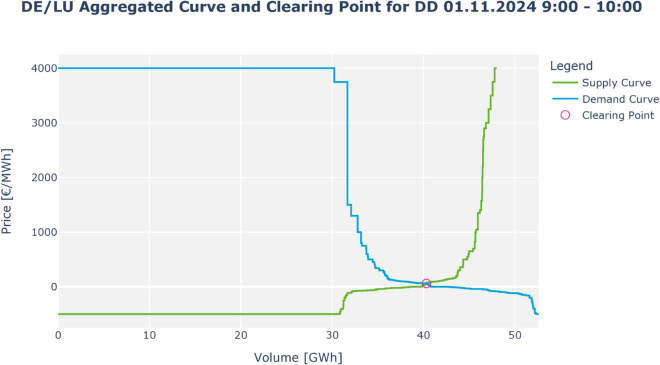
Market clearing in the day-ahead market. Sketch of the merit order curves for offers and demands being matched by the Simulation Facility in this analysis.

**Fig 10:**
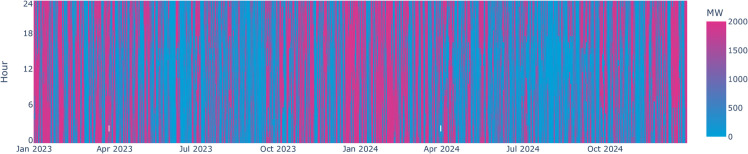
Wind availability profile. At hourly resolution based on wind speeds as measured in the Kriegers Flak offshore wind farm and scaled to the new offshore hub.

**Fig 11:**
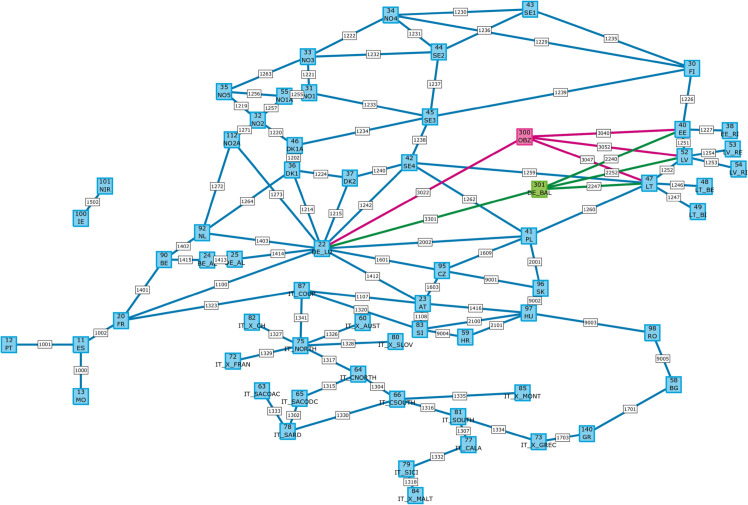
Market topology. Graphical representation of simulated bidding zones with identifiers. The added offshore hub is highlighted along with the utilized topologies per scenario. Red for the OBZ radial connection of scenario one and two as well as hybrid connection of scenario three. Green for the DE_BAL parallel interconnector of scenario two.
